# The Effects of Constraint-Induced Movement Therapy on Functions of Cerebral Palsy Children 

**Published:** 2018

**Authors:** Ali Reza JAMALI, Malek AMINI

**Affiliations:** 1 Department of Occupational Therapy, School of Rehabilitation Sciences, Iran University of Medical Sciences, Tehran, Iran.

**Keywords:** Constraint-induced movement therapy, Cerebral palsy, Hemiplegia, Rehabilitation; Systematic review

## Abstract

**Objectives:**

Constraint-Induced Movement Therapy (CIMT) is an intervention method that can enhance cerebral palsy (CP) children’s hand function. CP is a pervasive and common disorder which affects many aspects of a child life. Hemiplegic CP affects one side of a child’s hand and has great effect on child’s independence. We investigated the CIMT’s studies conducted in Iran, and indicated the effectiveness of CIMT on duration and children age?

**Materials & Methods:**

This systematic review was conducted using the electronic databases such as Medline PubMed, CINAHL, etc. performed from 1990 to 2016. Iranian and foreigner famous journals in the fields of pediatrics such as Iranian Journal of Pediatrics (IJP), Iranian Rehabilitation Journal (IRJ) and Google scholar with some specific keywords such as CP, CIMT, and occupational therapy were searched.

**Results:**

Overall, 43 articles were found, from which, 28 articles were removed because of lack of relevancy. Ten article were omitted because of duplication and exclusion criteria, so finally 15 articles were included.

**Conclusion:**

CIMT is effective compared to no intervention but there are some inconsistencies regarding some parts of CIMT effectiveness such as its effectiveness on muscle tone and protective extension.

## Introduction

Cerebral palsy (CP) is one of the primary causes of childhood disability, and it has a deep effect on physical and social functions (1). Although in recent years a lot of progress in prenatal care, genetic screening, birth control methods, NICU, and advanced centers for children care has emerged, nevertheless the prevalence of CP has remained stable between 1/5 and 2/5 per thousand live births. According to American Statistics Center, CP’s prevalence is between 2.6 to 2.9 per thousand live births (2). Low birth weight or premature children are susceptible to the CP. This children’s brain suffers from periventricular leukomalacia and intraventricular hemorrhage (3, 4). 

Since the major skill-based activities and physical-based activities accomplished outside, need physical abilities so children with CP have fewer abilities to participate in such activities because of their psychical problems (5). These problems even affect children daily routine occupational performance, quality of life and, societal participation (6). We have different kinds of interventions for CP children such as upper limb splinting, virtual reality, kinesio taping, constraint-induced movement therapy (CIMT) and traditional techniques like Bobath technics (7-10).

In this study, articles in the field of CIMT for hemiplegic CP children were systematically reviewed. Although the prevalence of this disability is different in parts of the world, spastic hemiplegia is the most common subtypes of CP (11). Cortical and additionally subcortical lesions caused by asymmetrical periventricular leukomalacia, middle cerebral artery stroke, or intraventricular hemorrhages, happened within motor areas of the contralateral hemisphere to the affected limb are the main causes of this type of CP (12). Children with hemiplegic CP are experiencing such problems related to their upper extremity more than other parts of the body. Problems such as difficulty performing intricate movements, weak grasping ability, hypertonia, changed proprioception and decreased selective motor control (13). These children not only feel limitation in their capacity but also tend to limit the affected limb usage in daily routines activities. The desire for less use of affected limb in developing children is called developmental disregard (14). Developmental disregard can be seen as inability to use hand or affected limb potentials in daily routines activities. It generally has been compared with learned nonuse, which is a phenomenon can occur after stroke (15, 16). 

CIMT is a deviation from traditional treatments, used to treat hemiplegia. Its aim is to stimulate the functional use of the affected limb and reverse the process developmental is disregard (17). In this method, the unaffected or less affected limb is restrained, so the person has to use the affected limb. This method has risen up out of the intersection of behavioral brain research/learning hypothesis and disclosures in neuroscience with respect to neuroplasticity. CIMT is a kind of paradigm shift in rehabilitation of central nervous system injuries. It changes the paradigm from emphasis on compensatory skills to a desire for partial restoration (18). CIMT is the most convincing clinical treatment to improve sensory and mobility functions in hemiplegic CP children (13, 19). Two possible mechanisms may lead to more use of the affected limb (Overcoming developmental disregard). These two are a) Overcoming the learned non-use of the more affected arm (for example increased use of the more affected arm) and b) use-dependent cortical reorganization. By using Trans cranial Magnetic Stimulation (TMS), motor cortex mapping before and after CIMT were studied and the increase of motor output area size and MEP amplitudes were noticed (20). It shows enhanced neuronal excitability in the damaged hemisphere and the target muscles. With the use of FMRI8 activation of the motor cortex changes after CIMT (20).

CIMT drove from fundamental researches on monkeys (21). Traditionally in CIMT, the less affected or non-affected hand is restrained for 90% of the day. During this period the affected limb has to perform everyday activities (22). Difficulties of CIMT traditional protocol leads to establishment of new protocols with different training programs. One of these protocols is modified CIMT (mCIMT) (23). In CIMT treatment sessions lasts for 30 min and for ten weeks conducted three times per week. mCIMT includes three basic components of CIMT. These components are constrained, repeated practice and using behavioral techniques such as shaping (24). 

Three therapeutic protocols are most widely implemented in interventions. The first is derived from the work that determined this protocol for an eight-week intervention period. During these eight weeks, the child has to participate once a week in therapeutic sessions and practice 2 h a day in a structured fashion. A five-week intervention and the child’s hand must be restrained 8 h a day. In addition, the child must practice 2 h a day with his parents (25). Therapeutic interventions are given once or twice a week. Appropriate intervention time is two weeks and during each week the children practice 5 d and six hours a day in a group (25). However, we still do not know the optimal constrain duration for the best outcome, the best type of restriction and the best period of clinical training. For example, more studies need to be done to understand at what age the CIMT has the greatest influence on child’s performance (20). CP children treated by CIMT have different abilities on the level of performances and restrictions. It has to be determined how effective the intervention is on each of these children.

Because of the recent popularity of this therapy for upper limb movement restrictions of CP children (18, 26, 27), more research is needed to justify more intensive treatments such as CIMT (28). This study was done with the aim of a systematic review of the CIMT procedure between CP children groups in Iran.

## Materials & Methods

This study was a systematic evidence-based study. Searches was performed from 1990 to 2016. Following sources were used for data gathering.

**1. Electronic databases:** Medline PubMed, CINAHL, OVID Medline, Google Scholar, CINAHL Plus with Full Text, Cochrane databases of systematic reviews, ProQuest, Up to Date, Web of Science, OT search, OT direct, Pedro, SID, Magiran, IRAN MEDEX, MEDLIB and Iran doc. 

**2. Iranian and foreigner famous journal in the fields of pediatrics: **Iranian Journal of Pediatrics (IJP), Iranian Rehabilitation Journal (IRJ), Iranian Journal of Child Neurology (IJCN), Archive Physical Medicine and Rehabilitation, Developmental Medicine, Child Neurology, physical and occupational therapy in pediatrics, American journal of occupational therapy, etc. 

With the help of MESH, we used following keywords for searching in mentioned databases. The main goal of this study was to determine the articles with CIMT intervention. Main keywords for search were CP, CIMT, Iranian CP children, constraint-induced movement therapy, OT, physical therapy, rehabilitation, Intensive intervention and mCIMT.

The inclusion criteria were applied as follows: 1) Articles that are about Iranian CP children 2) All articles since 1990 till 2016 3) Published in full text 4) Published in English or Persian 4) Contains CIMT intervention. The review included interventional studies.

## Results

Overall, 43 articles were found, because of lack of relevancy and other issues the 18 articles were removed. Ten articles were omitted based on duplication and exclusion criteria, so finally 15 articles were included (Table 1).

**Table 1:** The summary of the results and methodology of the studies used in this study

	Authors	Year	Title	Method and protocol	Procedure	Outcome measures	Results	Conclusion
1	Rostami et al.(29)	2012	Eﬀect of treatment environment on modified constraint-inducedmovement therapy results in children with spastic hemiplegic cerebralpalsy: a randomized controlled trial	Randomized controlled trial (RCT) mCIMT	15 h of modified CIMT, three times/week for 10 sessions every other day Restriction: splint	**A**: upper limb coordination and upper limb speed and dexterity **B**: amount of use and quality of movement	All variables changes were significant. Include: upper limb coordination, upper limb speed, and dexterity, amount of use, quality of movement	Modified CIMT is eﬀective in improving upper limb function in spastic hemiplegic children.
2	*Hosseini* et al. (30)	2012	Effectiveness of ICF-based modified constraint-induced movement therapy on hand functions in children with hemiplegic cerebral palsy	Single subject (SS design) mCIMT	2 groups First group: conventional OT interventions Second group: 6H of mCIMT during 10 d Restriction: splint	**A**: bimanual coordination, upper extremity coordination, dexterity and, visual motor control **B**: dexterity ***C***: muscle tone ***D:*** ROM ***E:*** Caregiver perception	All variables changes were significant. Include: 2 point discrimination, PROM of wrist, bimanual coordination, dexterity, Caregiver perception, muscle tone	Implementing adapted CIMT through a child-friendly approach was proved to improve hand functions and activities of daily living.
3	Sabour et al. (31)	2013	The effect of combination of constraint-induced movement therapy with bimanual intensive therapy on upper limb function of children with hemiplegic cerebral palsy	RCT CIMT	2 groups Control group: OT interventions Intervention group: CIMT and BIM for 10 d for 45 min Restriction: sling	***A: ***for UE function, ***B:*** for muscle tone	UE function changes were significant but muscle tone didn’t change significantly.	The findings suggest that combination of CIMT and bimanual intensive therapy improved upper limb Function in the hemiplegic CP children.
4	Hosseini et al. (32)	2011	Effect of mCIMT on weight bearing and protective extension in hemiplegic CP children	Clinical trial mCIMT	2 groups Control group: OT interventions Interventions group: OT interventions plus 45m mCIMT for 6 wk 3 sessions per week Restriction: splint	***A***: weight bearing and protective extension	Weight-bearing changes were significant but a protective extension change was not.	mCIMT had effect on weighing bearing but it had no effect on protective extension.
5	Rostami et al.(33)	2010	Study of treatment environment effect on CIMT intervention outcome in hemiplegic CP children	RCT CIMT	Two groups CIMT for 10d of 3 wk for 1/5H Restriction: splint	***A:*** UE coordination, speed and skill ***B:*** quantity and quality of the motion	All variables changes were significant. Include: UE coordination, speed and skill, quantity and quality of the motion	Hand function improved in children with hemiplegic CP and better improvements at home shows enhancement of learning process and practice at familiar condition and environment.
6	Rostami et al. (34)	2011	Comparison of virtual reality technique and CIMT on upper extremity of hemiplegic CP children	RCT CIMT	3 groups 1/5H of every other day and for 4 wk. Interventions were virtual reality technique and CIMT Restriction: splint	***A:*** for speed and skill ***B:*** for quantity and quality of the motion	All variables changes were significant. Include: speed and skill, quantity and quality of the motion	Base on this study results, virtual reality technique, and CIMT are alternative to each other for improvement of upper extremity function in hemiplegic CP children
7	Garib M et al. (35)	2010	Effect of mCIMT on quality of affected upper extremity in hemiplegic CP children	SS design mCIMT	2 groups Control group: OT interventions Intervention group: OT interventions plus 3H of mCIMT for 6 wk Restriction: splint	***A***: grasp, WB, protective extension, separated motions	All variables changes were significant. Include: grasp, WB, protective extension, separated motion	This study showed that mCIMT is more effective on quality of upper extremity grasp capability.
8	*Rostami, *et al. (36)	2012	Efficacy of combined virtual reality with constraint-induced movementtherapy on upper limb function of children with hemiparetic cerebral palsy	SS design CIMT	4 groups of CIMT, VR, CIMT+VR, and controls. Subjects in experimental groups participated in 1/5 H therapeutic sessions every other day during a four-week period Restriction: splint	***A:*** for quantity and quality of the motion, ***B:*** Test for speed and skill	All variables changes were significant. Include: quantity and quality of the motion speed and skill	Incorporating VR and CIMT may improve upper limb functioning of children withhemiparetic cerebral palsy.
9	*Kavoosipor, *et al. (37)	2010	Effects of constraint-induced movement therapy on improving in-handmanipulation skills of hemiplegic hand: A single-subject experimental study	SS design CIMT	21d of intervention with CIMT protocol plus30 min group program Restriction: splint	***A:*** for quality of finger to palm, palm to finger, simple shift, simple rotation, complex shift and complex rotation transfer. Frequency offinger to palm and palm to finger transfer. Rate of simple shift, complex rotation, and complex shift. Duration of simple rotation.	These were significant immediately after intervention: quality of Palm to finger and Complex shift transfer and Duration of simple rotation	A client-centered intervention will facilitate the use and quality of finger and hand motion. Moreover, group activities can encourage clients to participate more and better in therapy.
10	*Akbar Fahimi* et al.(38)	2011	Emotional Problems After UsingConstraint Induced Movement Therapyin Children with Hemiplegic CerebralPalsy	RCT CIMT	CIMT 6 h/day for 8 wkconsecutive d Restriction: splint	***A:*** behavioral assessment	Statistical analysis showed no significant difference intotal score and subscales scores of SDQ between two groups.	Using CIMT in children with hemiplegic CP could result in more usage of affected limb without any Behavior problems, especially emotional problems.
11	Hosseini et al.(39)	2010	Effect of Child-friendly Constraint-Induced Movement Therapy onunimanual and bimanual function in hemiplegia	SS design CIMT	Two groups of CIMT and conventionaltherapy. Intervention at CIMT was done six h every day, for 10 d, whereas another group receivedconventional occupational therapy. Restriction: splint	***A:***bilateral coordination,upper limb coordination, and upper limb dexterity and unimanual function ***B:*** Caregivers’ perception ***C:*** test for hand function	Changes of Unimanual function, Jebson-Taylor test, Dexterity, Bimanual function, Bilateral coordination, Caregivers’ perception (How Much), Caregivers’ perception (How Well) were significant but Bimanual coordination changes were not.	Child-friendly CIMT has fairly good effects on unimanual function and some variables of bimanual function of children with hemiplegia.
12	Kavousipor et al.(41)	2012	Can constraint induced movement therapy improve In-HandManipulation skills: a single subject design	SS design mCIMT	21 dof intervention 30 min every Day at clinic 6 H at home Restriction: splint	***A: ***quality of finger to palm, palm to finger, simple shift, simple rotation, complex shift and complex rotation transfer. Frequency offinger to palm and palm to finger transfer. Rate of simple shift, complex rotation and complex shift. Duration of simple rotation.	These variable changes were significant: Quality of palm to finger transfer. Frequency of palm to finger transfer.Quality of simple shift. Rate of simple shift.Quality of complex shift. Rate of complex shift. Duration of simplerotation. quality of complex	A client center intervention will facilitate the use and quality of fingers and hand motion.Also, a group activity can motivate participants to participate more and better.
13	Sabour et al.(41)	2013	Comparison of combination of CIMT and BIM training withCIMT alone on fine Motor Skills of children with Hemiplegic Cerebral Palsy	RCT CIMT	2 groups CIMT and BIM training and CIMT alone First group: 3H of CIMT and 3HBIM Second group: 6H of CIMT BOTH for 10 d Restriction: Sling	***A:*** testof hand function to evaluate the unilateral performance of theaffected limb, ***B:*** bilateral coordination,upper limb coordination, and upper limb dexterityand speed, ***C:***scale for muscle tone	These variables changes were significant: Fine motor skills , bilateral function domain of Bruininks –Oseretsky and Jobson-Taylor and Bruininks-Oseretsky test items. But muscle tone didn’t significantly change	Results showed that these two treatment approaches improved fine motor skills in the hemiplegic children with cerebral palsy. Therefore, it is suggested to use a combination of CIMT and BIM training instead of CIMT alone in order to make the tasks more attractive and easier for the children
14	Abootalebiet al. (42)	2010	The effects of "Constraint-Induced Movement Therapy" on fine motor skills in children with hemiplegic cerebral palsy	RCT mCIMT	2 groups intensive occupational therapy program for bothfive hours per day for 21 d intervention group: 5H of CIMT for 21 d restriction: sling	***A:*** fine motor skills, ***B:*** muscle tone ***C:*** was for neurofeedback	Peabody developmental motor scales changes were significant. But changes of modified Ashworth scale, H reflexand H/M ratio was not.	Results suggest that the use of CIMT needs to more studies and should be considered experimental in children with hemiplegic CP
15	Garib M et al. (43)	2011	Effect of mCIMT on grasp quality in hemiplegic CP children	RCT mCIMT	2 groups occupational therapy program for both for 6Wintervention group: 3H CIMT for 6W Restriction: splint	***A:*** for grasp quality	grasp quality significantly improved	The results of this study showed that mCIMT has effect on grasp quality in hemiplegic CP children

## Discussion

The aim of this study was to investigate the CIMT interventions carried out in Iran. CIMT is an effective intervention method for CP children. Eight studies had used traditional CIMT and 7 studies had used mCIMT protocol.

CIMT for CP children has little to do with age. The age range for CIMT in the studies is between two years to 14 years and in all of them, CIMT had acceptable results. The therapeutic effect of CIMT was not age-related (44). They also confirmed the results of sung and DeLuca’s study (2, 3). There were no differences between boys and girls for this therapy and CIMT was equally effective for both genders. Gender was reported as an ineffective factor in CIMT too (21, 45). 

Articles reviewed in the study had used only two kinds of restrictions. Most of them had used a splint for restriction (12 of them) and three of them had used sling for restriction. For this reason, maybe the use of splints and slings are easier for children. Other kinds of restriction were reported too. Restrictions such as Short arm casts and Long arm casts, holding child’s hand, using a glove or mitt and Slings (20). CIMT effect on the left or right side is the same because no study mentioned to affected side.

For concerning the effect of CIMT on muscle tone the result of four articles about the impact of CIMT on muscle tone was inconsistent. CIMT had an influence on muscle tone (30). CIMT was considered as an ineffective method on muscle tone (31, 41, 42). CIMT was considered as an ineffective way of reducing muscle tone (46). However, this study did not find definite conclusion about the impact of CIMT on muscle tone. This issue requires further studies in the future.

CIMT has a good effect on protective extension. It was not effective on protective extension (32); however, it was effective on protective extension (35). CIMT was effective on protective extension (20), however, in another study; CIMT was not effective on protective extension (47). In this case, literature are not unified and more studies are needed.

There were no significant adverse effects for CIMT in the studies. Nevertheless, early implementation of CIMT for children who are in the stages of development of bilateral hand can cause a negative effect on the growth of bilateral hand development. Therefore, CIMT should be used with caution for children under twelve months (48). In addition, restriction of the non-affected hand for a long time (e.g. plastering) had negative effects on the development of motor skills (46). 

CIMT was examined efficacy on children’s participation in activity of daily living, and no studies had measured CIMT effect on occupational performance (30). Improvement of sub-skills do not always accompany improvement of daily living activities occupational performance, so future studies would also consider this issue. Because the ability of an intervention to improve the level of independence is undeniably important.

**In conclusion, **in recent years CIMT has attracted much attention in Iran and different studies with different methods have been conducted. Researchers have used various restriction time and different outcome measures. There are some inconsistencies in some aspects of CIMT effectiveness such as muscle tone and protective extension. These areas need future research. In addition, more studies are needed to investigate negative effects of CIMT from physical and social aspects. 

Follow-ups are an important aspect of rehabilitation intervention. Less than half of the studies had included follow-up in their method. In the end, if we consider hands as brain’s tool for independence in everyday activities more attention has to be paid on follow-ups and other occupational aspects.
